# Supplementary figures and images for: Prediction of microbe-drug associations using a CNN-Bernoulli random forest model
Source: PeerJ. 2025 Aug 5;13:e19637. doi: 10.7717/peerj.19637 (PMC12333605; doi:10.7717/peerj.19637)

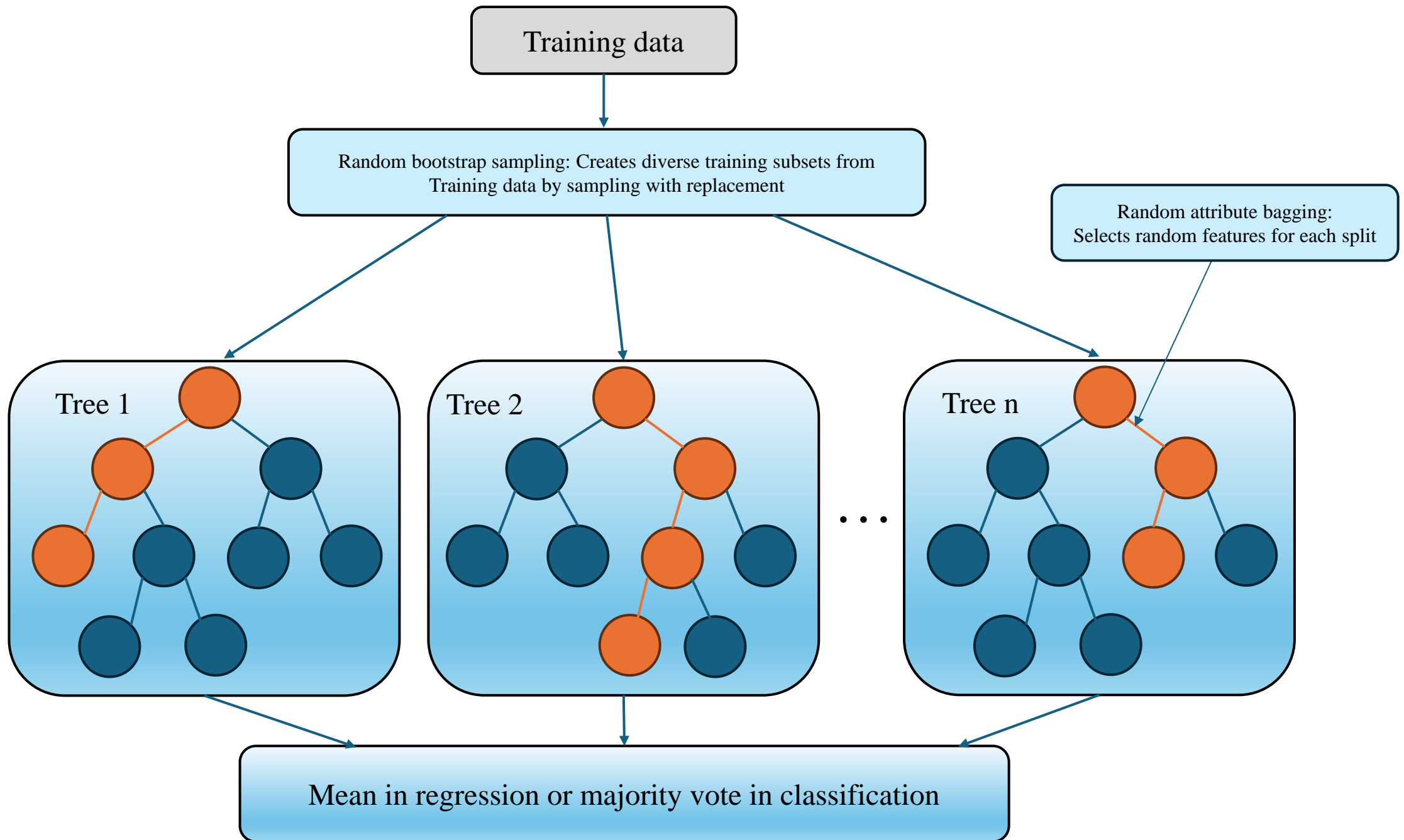

Supplement: Supplemental Information 3 [file peerj-13-19637-s003.pdf]

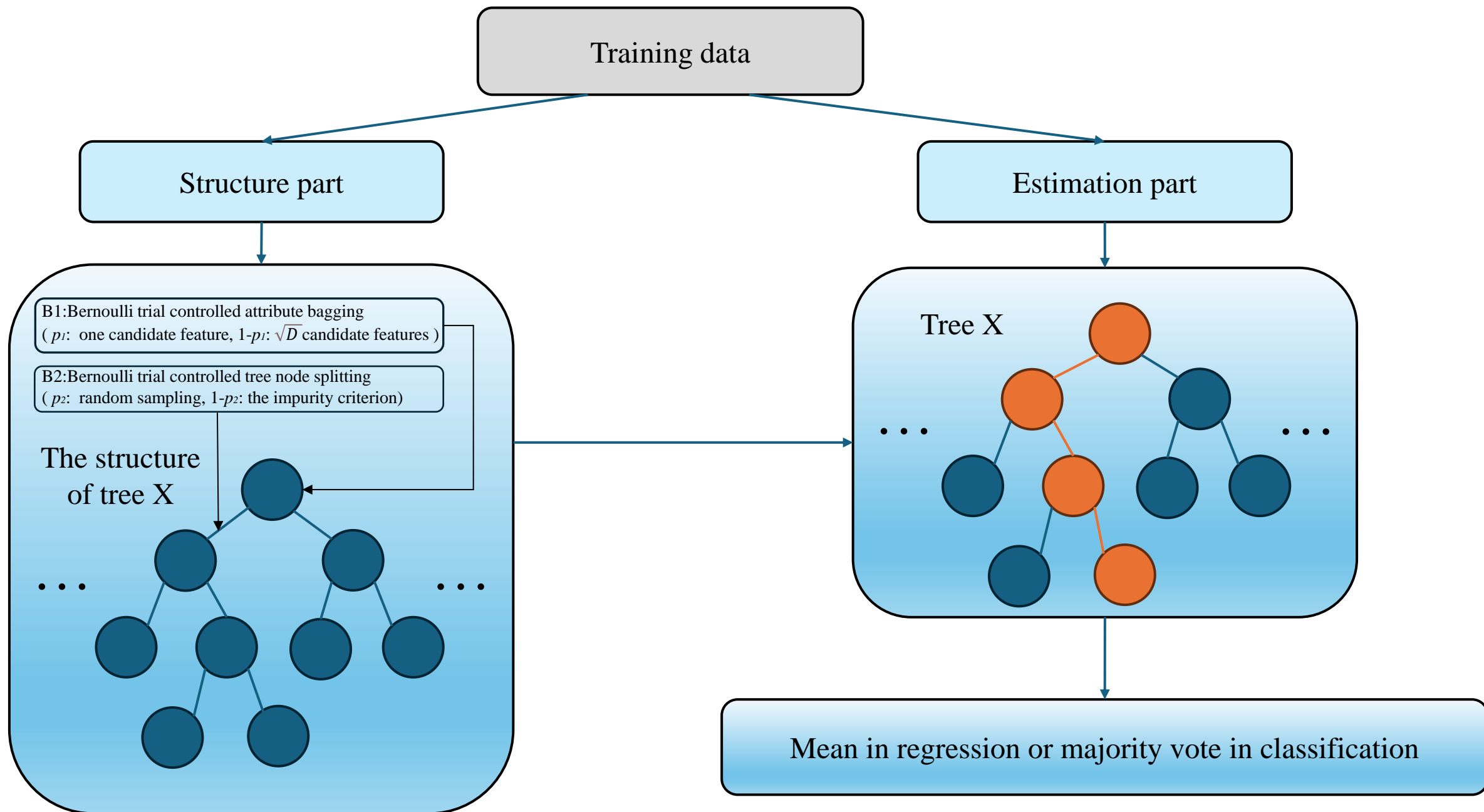

Supplement: Supplemental Information 4 [file peerj-13-19637-s004.pdf]

Five-Fold Validation Performance on MADA Dataset

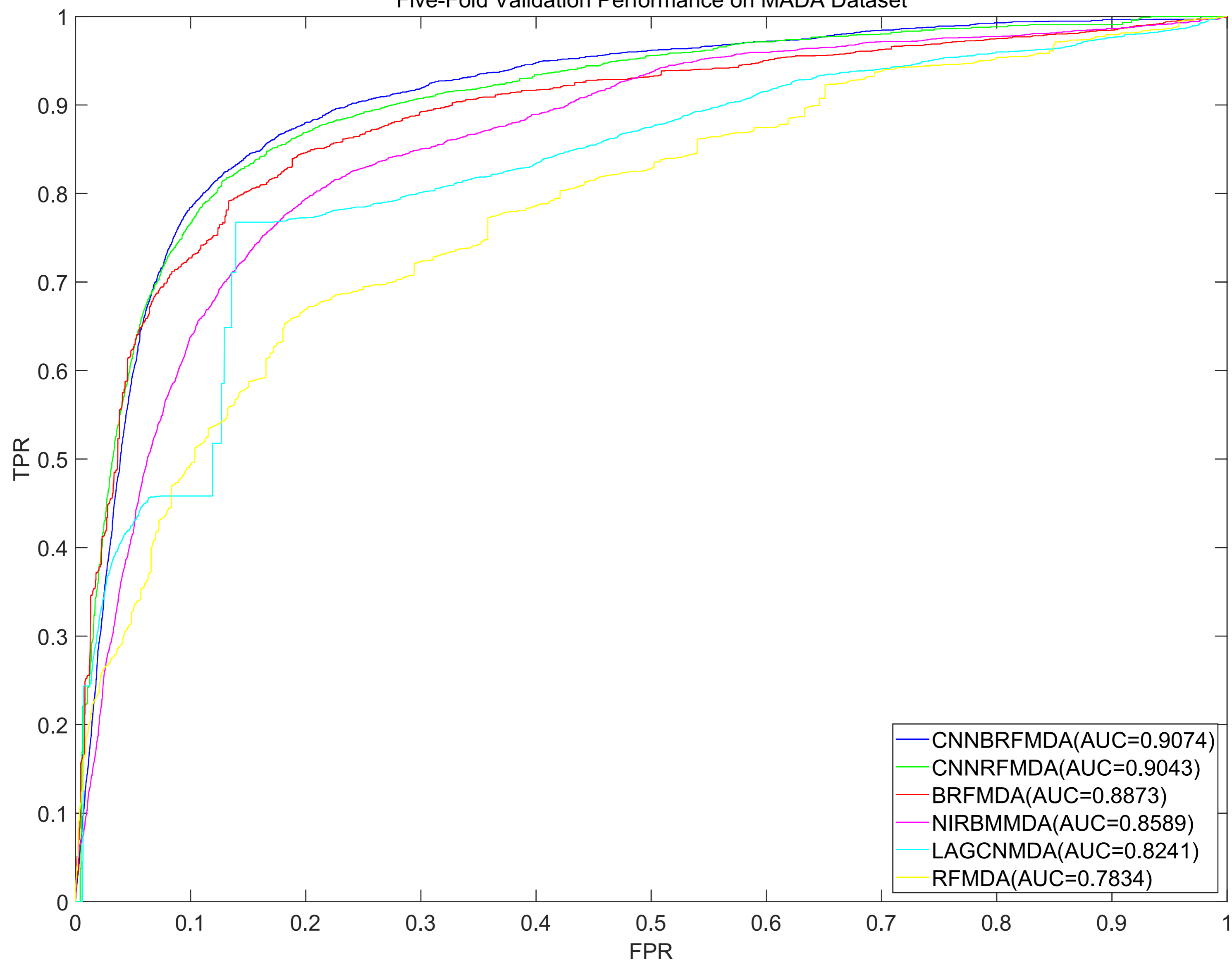

Supplement: Supplemental Information 5 [file peerj-13-19637-s005.pdf]

Five-Fold Validation Performance on abiofilm Dataset

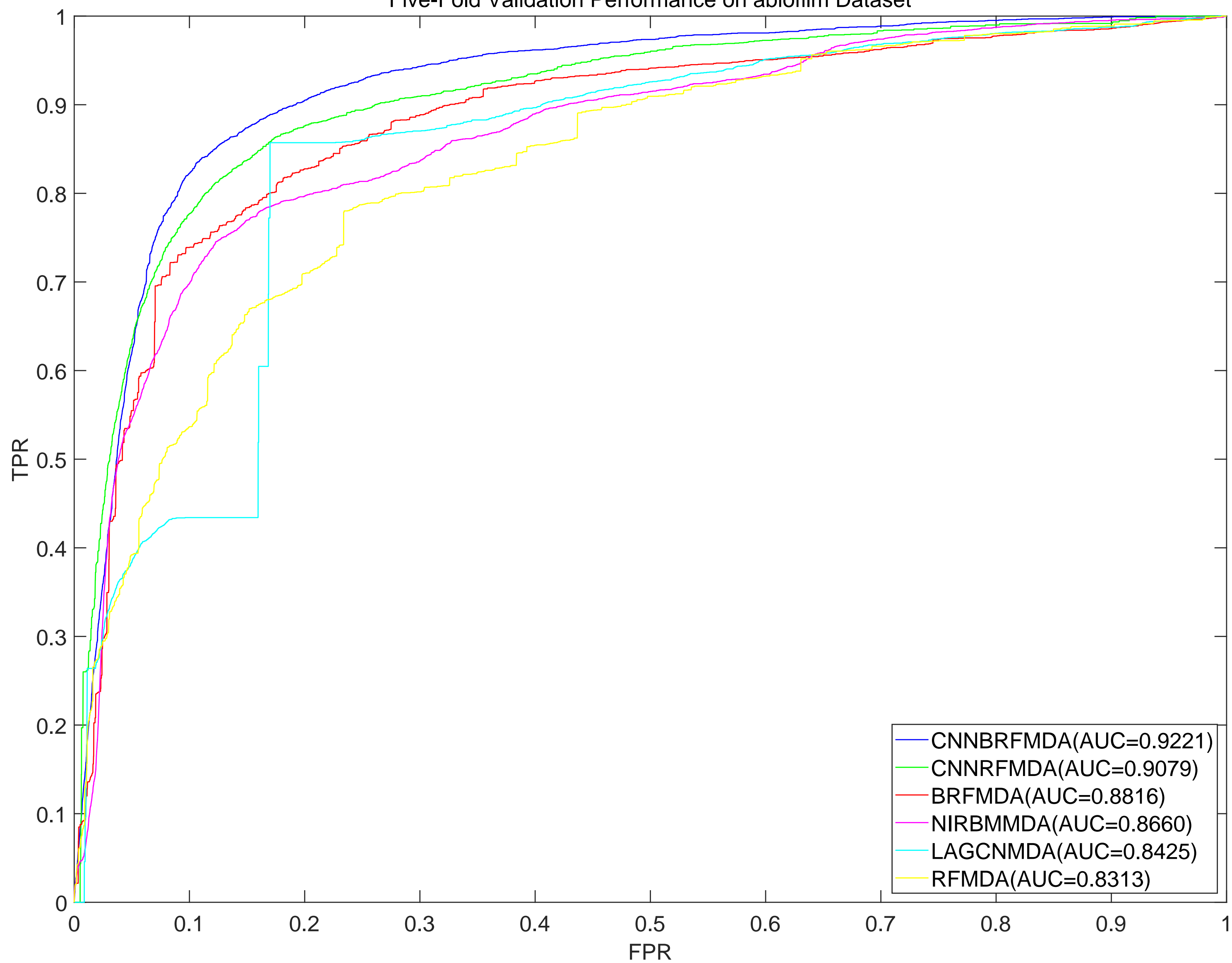

Supplement: Supplemental Information 6 [file peerj-13-19637-s006.pdf]

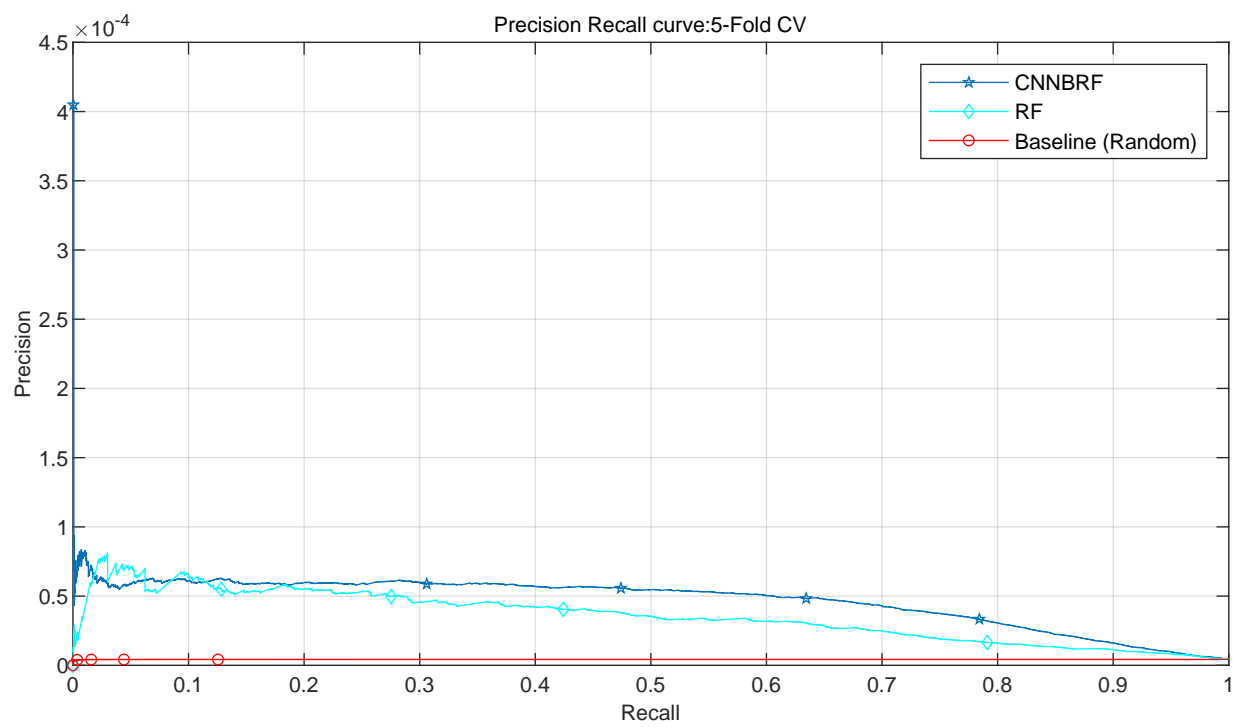

Supplement: Supplemental Information 7 [file peerj-13-19637-s007.pdf]

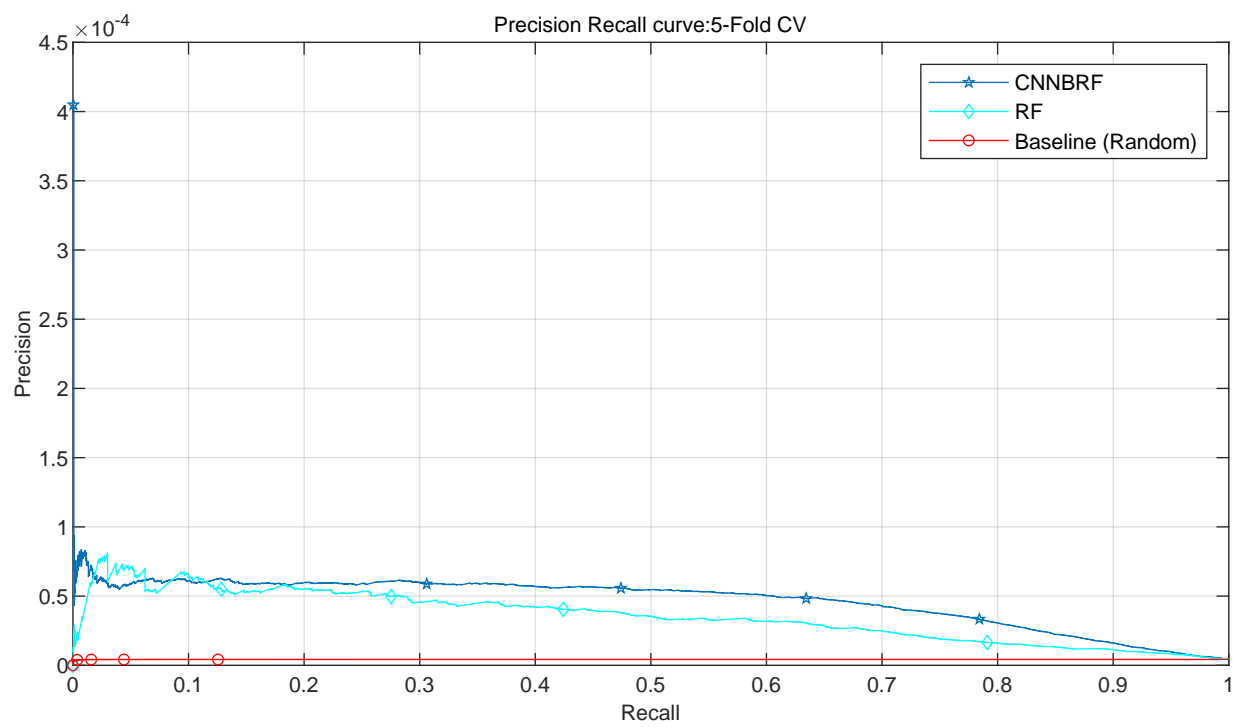

Supplement: Supplemental Information 8 [file peerj-13-19637-s008.pdf]

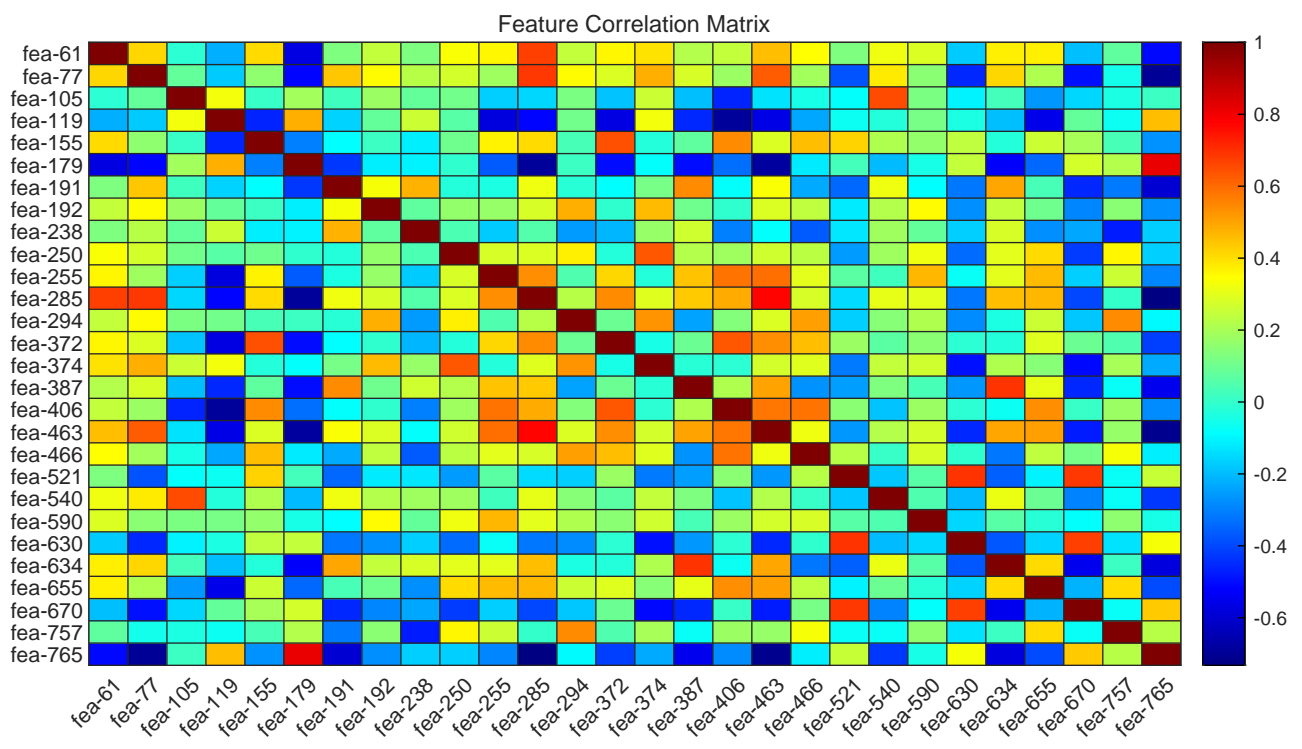

Supplement: Supplemental Information 9 [file peerj-13-19637-s009.pdf]
