# Supplementary material for: Prediction of microbe-drug associations using a CNN-Bernoulli random forest model
Source: PeerJ. 2025 Aug 5;13:e19637. doi: 10.7717/peerj.19637 (PMC12333605; doi:10.7717/peerj.19637)
Supplement: Supplemental Information 10 [file peerj-13-19637-s010.docx]

| **Prediction model** | **AUC** | **Standard deviation** |
| --- | --- | --- |
| CNNBRFMDA | 0.9017 | 0.0032 |
| CNNRF | 0.9008 | 0.0025 |
| BRF | 0.8836 | 0.0025 |
| NIRBM | 0.8691 | 0.0047 |
| RF | 0.8296 | 0.0265 |
| LAGCN | 0.8279 | 0.0019 |
| logistic regression | 0.8257 | 0.0026 |
| k-nearest neighbors | 0.7900 | 0.0023 |
