# Supplementary material for: Prediction of microbe-drug associations using a CNN-Bernoulli random forest model
Source: PeerJ. 2025 Aug 5;13:e19637. doi: 10.7717/peerj.19637 (PMC12333605; doi:10.7717/peerj.19637)
Supplement: Supplemental Information 11 [file peerj-13-19637-s011.docx]

| **Prediction model** | **AUC** | **Standard deviation** |
| --- | --- | --- |
| CNNBRFMDA | 0.9146 | 0.0041 |
| CNNRF | 0.9118 | 0.0026 |
| BRF | 0.8847 | 0.0030 |
| NIRBM | 0.8624 | 0.0034 |
| LAGCN | 0.8430 | 0.0020 |
| logistic regression | 0.8410 | 0.0011 |
| RF | 0.8076 | 0.0337 |
| k-nearest neighbors | 0.7606 | 0.0023 |
