# Supplementary material for: Prediction of microbe-drug associations using a CNN-Bernoulli random forest model
Source: PeerJ. 2025 Aug 5;13:e19637. doi: 10.7717/peerj.19637 (PMC12333605; doi:10.7717/peerj.19637)
Supplement: Supplemental Information 12 [file peerj-13-19637-s012.docx]

| **Prediction model** | **AUC** | **Standard deviation** |
| --- | --- | --- |
| CNNBRF-epoch10 | 0.8939 | 0.0064 |
| CNNBRF-epoch20 | 0.9017 | 0.0032 |
| CNNBRF-epoch50 | 0.9079 | 0.0027 |
| CNNBRF-epoch100 | 0.9081 | 0.0043 |
