# Supplementary material for: Prediction of microbe-drug associations using a CNN-Bernoulli random forest model
Source: PeerJ. 2025 Aug 5;13:e19637. doi: 10.7717/peerj.19637 (PMC12333605; doi:10.7717/peerj.19637)
Supplement: Supplemental Information 13 [file peerj-13-19637-s013.docx]

| **Prediction model** | **AUC** | **Standard deviation** |
| --- | --- | --- |
| CNNBRF-epoch10 | 0.9121 | 0.0041 |
| CNNBRF-epoch20 | 0.9146 | 0.0041 |
| CNNBRF-epoch50 | 0.9182 | 0.0057 |
| CNNBRF-epoch100 | 0.9197 | 0.0051 |
