# Supplementary material for: Prediction of microbe-drug associations using a CNN-Bernoulli random forest model
Source: PeerJ. 2025 Aug 5;13:e19637. doi: 10.7717/peerj.19637 (PMC12333605; doi:10.7717/peerj.19637)
Supplement: Supplemental Information 14 [file peerj-13-19637-s014.docx]

| **Microbe name** | **Evidence** | **Microbe name** | **Evidence** |
| --- | --- | --- | --- |
| Escherichia coli | PMID:33106267 | Mixed Culture of bacteria and fungus | Unconfirmed |
| Enterococcus faecalis | PMID:25698261 | Staphylococcus epidermis | PMID:10632381 |
| Bacillus subtilis | PMID:30758259 | Burkholderia multivorans | PMID:34524889 |
| Proteus vulgaris | PMID:11418511 | Pseudoalteromonas sp. | PMID:31137680 |
| Proteus mirabilis | PMID:21437168 | Pseudomonas chlororaphis | Unconfirmed |
| Mycobacterium avium | PMID: 8239587 | Eikenella corrodens | PMID: 16875802 |
| Morganella morganii | PMID:29942700 | Porphyromonas gingivalis | PMID: 15231772 |
| Pichia anomala | Unconfirmed | Flavobacterium psychrophilum | Unconfirmed |
| Candida albicans | PMID:31471074 | Streptococcus pneumoniae | PMID:15155208 |
| Streptococcus mutans | PMID:19109335 | Bacillus spp. | PMID:26358183 |
| Staphylococcus epidermidis | PMID:30760047 | Serratia marcescens | PMID:23751969 |
| Staphylococcus saprophyticus | PMID:21905846 | Campylobacter jejuni | PMID:11920303 |
| Shigella sonneii | PMID:33993224 | Pseudomonas japonica | PMID:30550842 |
| Salmonella enterica | PMID:32747937 | Micrococcus luteus | PMID:3032098 |
| Vibrio parahaemolyticus | PMID:34351514 | Vibrio cholerae | PMID:31664493 |
| Streptococcus epidermidis | PMID:10632381 | Providencia stuartii | PMID:1337751 |
| Burkholderia pseudomallei | PMID:27936915 | Burkholderia thailandensis | PMID:23872555 |
| Erwinia carotovora | Unconfirmed | Staphylococcus aureus | PMID:32882442 |
| Burkholderia cenocepacia | PMID:25267676 | Agrobacterium tumefaciens | Unconfirmed |
| Streptococcus mitis | PMID:10348783 | Staphylococcus haemolyticus | PMID: 37265496 |
| Candida dubliniensis | PMID:30781782 | Staphylococcus equorum | PMID24142792 |
| Vibrio harveyi | PMID:27247095 | Staphylococcus capitis | Unconfirmed |
| Candida parapsilosis | Unconfirmed | Aeromonas hydrophila | PMID:21339408 |
| Klebsiella pneumoniae | PMID:27257956 | Burkholderia stabilis | PMID:15571853 |
| Burkholderia vietnamiensis | Unconfirmed | Yersinia enterocolitica | PMID:32889419 |
