# Supplementary material for: Prediction of microbe-drug associations using a CNN-Bernoulli random forest model
Source: PeerJ. 2025 Aug 5;13:e19637. doi: 10.7717/peerj.19637 (PMC12333605; doi:10.7717/peerj.19637)
Supplement: Supplemental Information 15 [file peerj-13-19637-s015.docx]

| **Microbe name** | **Evidence** | **Microbe name** | **Evidence** |
| --- | --- | --- | --- |
| Haemophilus influenzae | PMID: 31740553 | Enterococcus faecalis | PMID:31763048 |
| Candida albicans | PMID: 28409362 | Candida spp. | PMID:30768071 |
| Escherichia coli | PMID:31542319 | Mycobacterium tuberculosis | PMID:31414626 |
| Stenotrophomonas maltophilia | PMID: 27257956 | Klebsiella pneumoniae | PMID:27257956 |
| Bacillus subtilis | PMID: 30036828 | Proteus vulgaris | PMID:12482994 |
| Staphylococcus aureus | PMID:12654680 | Aquifex aeolicus | Unconfirmed |
| Streptococcus pneumoniae | PMID: 11249825 | Vibrio harveyi | Unconfirmed |
| Streptococcus pneumoniae serotype 4 | PMID: 22407042 | Yersinia enterocolitica | PMID:32648451 |
| Human immunodeficiency virus | PMID: 36041016 | Sphingomonas sp. Ibu-2 | Unconfirmed |
| Listeria monocytogenes | PMID:18299415 | Listeria ivanovii | PMID:36981047 |
| Streptococcus epidermidis | Unconfirmed | Hafnia alvei | PMID:32111359 |
| Human immunodeficiency virus 1 | PMID:18441333 | Candida tropicalis | PMID:20455400 |
| Bacillus licheniformis | Unconfirmed | Human herpesvirus 1 | PMID: 32406606 |
| Enteric bacteria and other eubacteria | Unconfirmed | Proteus mirabilis | PMID:32833846 |
| Clostridium perfringens | PMID:32707228 | Morganella morganii | PMID:29942700 |
| Coagulase- negative staphylococci | PMID:15211086 | Porphyromonas gingivalis | PMID:30048853 |
| Listeria monocytogenes serotype 4a | PMID: 28739228 | Halomonas pacifica | Unconfirmed |
| Streptococcus mutans | PMID:27550428 | Chromobacterium violaceum | PMID: 27747113 |
| Geobacillus kaustophilus | Unconfirmed | Enterobacter aerogenes | PMID:10428935 |
| Staphylococcus saprophyticus | PMID:24982521 | Vibrio vulnificus | PMID:12384368 |
| Human parainfluenza virus type 2 | Unconfirmed | Vibrio anguillarum | Unconfirmed |
| Plasmodium falciparum | PMID:15125930 | Eikenella corrodens | PMID:11897609 |
| Vibrio parahaemolyticus | PMID:31794931 | Dengue Virus Type 2 | Unconfirmed |
| Clostridium pasteurianum | Unconfirmed | Enterococcus gallinarum | PMID:21444994 |
| Influenza A virus | PMID:29540219 | Influenza B virus | PMID:34918028 |
